# Histologic subtyping affecting outcome of triple negative breast cancer: a large Sardinian population-based analysis

**DOI:** 10.1186/s12885-020-06998-9

**Published:** 2020-06-02

**Authors:** Francesca Sanges, Matteo Floris, Paolo Cossu-Rocca, Maria R. Muroni, Giovanna Pira, Silvana Anna Maria Urru, Renata Barrocu, Silvano Gallus, Cristina Bosetti, Maurizio D’Incalci, Alessandra Manca, Maria Gabriela Uras, Ricardo Medda, Elisabetta Sollai, Alma Murgia, Dolores Palmas, Francesco Atzori, Angelo Zinellu, Francesca Cambosu, Tiziana Moi, Massimo Ghiani, Vincenzo Marras, Maria Cristina Santona, Luisa Canu, Enrichetta Valle, Maria Giuseppina Sarobba, Daniela Onnis, Anna Asunis, Sergio Cossu, Sandra Orrù, Maria Rosaria De Miglio

**Affiliations:** 1 Department of Biomedical Sciences, University of Sassari, Sassari, Italy; 2 Biomedicine Sector, Center for Advanced Studies, Research and Development in Sardinia Technology Park Polaris, Cagliari, Italy; 3 Department of Medical, Surgical and Experimental Sciences, University of Sassari, Via P. Manzella, 4, 07100 Sassari, Italy; 4 Department of Diagnostic Services, “Giovanni Paolo II” Hospital, ASSL Olbia-ATS Sardegna, Olbia, Italy; 5 School of Hospital Pharmacy, University of Sassari, Sassari, Italy; 6 Department of Environmental Health Sciences, Istituto di Ricerche Farmacologiche Mario Negri IRCCS, Milan, Italy; 7 Department of Oncology, Istituto di Ricerche Farmacologiche Mario Negri IRCCS, Milan, Italy; 8 Department of Pathology, AOU Sassari, Sassari, Italy; 9 Department of Pathology, “A. Businco” Oncologic Hospital, ASL Cagliari, Cagliari, Italy; 10 Department of Medical Oncology, “A. Businco” Oncologic Hospital, ASL Cagliari, Cagliari, Italy; 11 Medical Oncology Unit, AOU, Cagliari, Italy; 12 Department of Pathology, ASSL Nuoro, Nuoro, Italy; 13 Department of Medical Oncology, ASSL Nuoro, Nuoro, Italy; 14 Department of Pathology, Brotzu Hospital, Cagliari, Italy

**Keywords:** Triple negative breast cancer, Clinico-pathological features, Prognosis, Histologic special type, Tumor size, Metastatic lymph node

## Abstract

**Background:**

Triple Negative breast cancer (TNBC) includes a heterogeneous group of tumors with different clinico-pathological features, molecular alterations and treatment responsivity. Our aim was to evaluate the clinico-pathological heterogeneity and prognostic significance of TNBC histologic variants, comparing “*special types*” to high-grade invasive breast carcinomas of no special type (IBC-NST).

**Methods:**

This study was performed on data obtained from *TNBC Database,* including pathological features and clinical records of 1009 TNBCs patients diagnosed between 1994 and 2015 in the four most important Oncology Units located in different hospitals in Sardinia, Italy. Kaplan-Meier analysis, log-rank test and multivariate Cox proportional-hazards regression were applied for overall survival (OS) and disease free survival (DFS) according to TNBC histologic types.

**Results:**

TNBC “*special types”* showed significant differences for several clinico-pathological features when compared to IBC-NST. We observed that in apocrine carcinomas as tumor size increased, the number of metastatic lymph nodes manifestly increased. Adenoid cystic carcinoma showed the smallest tumor size relative to IBC-NST. At five-year follow-up, OS was 92.1, 100.0, and 94.5% for patients with apocrine, adenoid cystic and medullary carcinoma, respectively; patients with lobular and metaplastic carcinoma showed the worst OS, with 79.7 and 84.3%, respectively. At ten-years, patients with adenoid cystic (100.0%) and medullary (94.5%) carcinoma showed a favourable prognosis, whereas patients with lobular carcinoma showed the worst prognosis (73.8%). TNBC medullary type was an independent prognostic factor for DFS compared to IBC-NST.

**Conclusions:**

Our study confirms that an accurate and reliable histopathologic definition of TNBC subtypes has a significant clinical utility and is effective in the therapeutic decision-making process, with the aim to develop innovative and personalized treatments.

## Background

Breast cancer (BC) is a heterogeneous disease, which encompasses various entities showing significant differences in morphologic and prognostic features, as well as in therapeutic options [1]. Recently, a marked molecular heterogeneity of breast cancer has also been demonstrated by gene expression profiling studies, which identified four major BC “*intrinsic*” subtypes, including luminal A, luminal B, HER2-enriched, and basal-like, showing variable biological, clinical behaviors and response to treatment [2, 3]. So far, invasive breast cancer has been classified according to histological features and immunohistochemical expression of estrogen receptors (ER), progesterone receptors (PR) and HER2 overexpression and/or *HER2* gene amplification [4]. Interestingly, BC subtyping by immunohistochemistry (IHC) is concordant with gene expression profiles, therefore having significant clinical utility [5, 6].

Particularly, ER/PR/HER2 negative immunostain defines the Triple Negative subtype, which accounts for 10 -20% of all invasive breast cancer types. TNBC is most prevalent in young women, < 50 years of age, showing aggressive clinical behavior, high histological grade and poor prognosis, and is responsible for about 25% of BC-related deaths. TNBC comprehends tumors with different clinico-pathological features and genetic-molecular alterations [7], and it is prevalently histological categorized as IBC-NST. Other histologic “*special types*” (HST), such as metaplastic, apocrine, lobular and adenoid cystic carcinomas, are still included among TNBC. These special phenotypes substantially differ in terms of biological behavior and clinical course [8]. Among TNBC histotypes, the historical morphological entity of medullary carcinoma previously considered as a specific BC special type in the category of “carcinoma with medullary features”, is no longer identified as a special type variant and has been recently reconsidered as IBC-NST with medullary pattern, rather than a distinct morphological subtype; moreover, medullary-type pattern is often associated with variable immunohistochemical expression of basal markers [9].

Recent studies evaluated the outcome of patients with TNBC special types showing that distinct prognostic implications derive from these morphologic features, highlighting that the identification of the special types has a significant clinical utility, and should be considered in therapeutic algorithms [10–14].

The aim of this study was to analyze a uniquely large cohort of TNBC patients enrolled in Sardinia, Italy, in order to evaluate the clinico-pathological heterogeneity, taking into account several parameters known for their prognostic and predictive roles, as well as highlighting the correlation between tumor size and lymph nodes status according to TNBC histologic subtypes. The prognostic significance of histologic special types compared with IBC-NST triple negative variants was also investigated.

## Methods

The study was conducted in accordance with the code of ethics of the World Medical Association (Declaration of Helsinki). The protocol was approved by the local research ethics committee of Sardinia Region (File number 224/CE/12); according to the Italian legislation on guidelines for the implementation of retrospective observational studies (G.U. n. 76, 31/03/2008) it renounced the written informed consent from patients. To protect patient confidentiality, only coded data without direct patient identifiers were collected. Information was obtained from retrospectively collected *TNBC Database* on all consecutive patients with Triple Negative breast cancer diagnosis surgically treated in the four most important Oncology Units located in different hospitals in Sardinia, from 1994 to 2015, as previously published [15]. Specifically, in the present study, a total of 1009 primary TNBC patients were recruited based on further revision and integrations of TNBC patients in our dynamic *TNBC Database*. Three experienced pathologists reviewed all tumors cases independently, and histologic subtyping has achieved according to current WHO classification [16].

### Immunohistochemical analysis

ER, PR, and HER2 immunohistochemical expression and/or *HER2* gene amplification, as defined by silver-enhanced “in situ” hybridization (SISH), established TNBC status. The IHC analysis was performed using specific antibodies against monoclonal rabbit ER antibody, Clone SP1 (Neomarker, Fremont, CA USA), monoclonal mouse PR antibody, Clone PgR 636 (DakoCytomation, Glostrup, Denmark). Moreover, Ki-67 and androgen receptors (AR), were also evaluated with monoclonal mouse Ki-67, clone MM1 (Leica Biosystems, Wetzlar, Germania) and mouse monoclonal AR, clone 2F12, (Novocastra, Dublin, OH, USA), respectively.

ER, and PR expression were positive if at least 1% immunostained tumor nuclei were detected in the sample, according to the American Society of Clinical Oncology/College of American Pathologists (ASCO/CAP) recommendations for immunohistochemical testing of hormone receptors in BC [17], whose criteria have recently been adopted by WHO classification [16].

AR expression was considered positive if at least 1% immunostained tumor nuclei were detected in the sample and categorized using semi-quantitative expression [18]. The Ki67 cut-off < 14, 15–30% and > 30% were based on results obtained in our previous study by Urru et al. 2018 [15]. HER2 protein expression was determined using FDA approved HercepTest™ K5206 (DakoCytomation) and evaluated according to the manufacturer’s instructions. *HER2* gene amplification was ascertained by ultra-View SISH Detection Kit (Ventana Medical Systems, Tucson, USA).

Given that the study included patients diagnosed over almost 20 years in different hospital centers, all surgical specimens of TNBC patients were reviewed independently by three experienced pathologists to achieve a consensus on morphologic criteria and to standardize the results according to the current guidelines recommendations for ER, PgR and HER2 immunohistochemistry [17]. Older cases, mainly from 1994 to 2005, and selected cases with not concordant morphology were immunostained again according to the protocol mentioned above.

### Patient information

*TNBC Database* includes personal and medical data collected from medical records of each TNBC patient. Specifically, it includes patients’ information on socio-demographic factors, anthropometric characteristics, obstetric and gynecologic features, lifestyle habits, family history of breast and other cancers, and various comorbidities. Moreover, pathologic assessments included information on tumor site and size, histologic type and grade, necrosis, lymphovascular invasion (LVI), AR and Ki67 expression, lymph node status, tumor grade, and pathologic TNM staging. Finally, tumor-infiltrating lymphocytes (TILs) were analyzed according to Denkert et al. criteria [19] and scored semi-quantitatively in two categories, low TILs (< 50.0%) and high TILs (> 50.0%), according to previous experiences [20]. Lymph node ratio was obtained from the number of metastatic lymph node divided by the number of lymph node evaluated by pathologist, and was categorized according to validated cut-off points, i.e. ≤ 0.2, 0.21–0.65, and ≥ 0.65 [21]. Finally, all clinical data of TNBC patients, including cancer treatments (surgery, radiotherapy and/or chemotherapy), TNBC recurrence, occurrence of other neoplasm(s), metastasis or death, were recorded. The baseline and follow-up data were defined as previously reported [15].

### Statistical analysis

Overall survival and disease-free survival were the main endpoints of this study. OS is defined as the time between the date at diagnosis and the date of death, and the DFS as the time from the date at diagnosis to the date of TNBC local recurrence, clinical metastatic diseases, occurrence of other primary cancers, death, or last follow-up visit, whichever occurred first.

Descriptive overall and subgroup analysis were carried out and differences in the basic characteristics and clinical parameters were analyzed using the Student t-test for normal distributed variables; Chi-Square test or Fisher’s exact test were used to test differences in frequencies, when appropriated, respectively. A violin plot analysis showed the distribution of tumor size according to histologic type. Mann-Whitney U test assessed the differences between histologic types. To study the relationship between tumor size (mm) and number of positive lymph nodes in different TNBC histological types, Spearman correlation and linear regression was used. In the linear regression model, the slope of the regression line indicates the mean increase in the number of positive nodes per millimeter of increase in tumor size. A *p* value for interaction was calculated to determine whether such effect varied significantly according to the histologic type. This analysis was limited to patients with at least five lymph nodes examined. All tests were two-sided, and a threshold of *p* < 0.05 was considered to identify statistically significant results. No statistical adjustment was made for multiple comparisons.

Multivariate hazard ratios (HRs) for recurrence and mortality, after 10 years of follow-up, according to TNBC histologic types and the corresponding 95% confidence intervals (CIs), were calculated by Cox proportional hazards model, corrected for age, tumor size and number of positive lymph node. Kaplan-Meier method and Log-Rank test were utilized to describe DFS and OS according to TNBC histologic types. Statistical analysis was performed in R packages "survminer" and "survival. The statistical significance was set-up at < 0.05.

## Results

### Clinico-pathologic features of TNBC histologic types

One thousand and nine TNBC patients were initially included in the study. Table 1 shows the pathological assessments including the overall distribution of TNBC according to histologic type. Tumors from 745 patients (78.1%) were classified as IBC-NST, 62 (6.5%) as lobular, 43 (4.5%) as apocrine, 42 (4.4%) as metaplastic, 39 (4.1%) as medullary; less common were other histologic types such as mixed IBC-NST and invasive lobular carcinoma (0.7%), papillary (0.7%), adenoid cystic (0.6%), mucinous (0.2%) and micropapillary (0.2%) carcinomas.

**Table 1:** Clinico-pathological features of “*Triple Negative*” breast cancer

Variables	TNBC n (%) (*n* = 1009) n (%)
**Age**	
< 35	47 (4.7)
35–49	263 (26.1)
50–69	503 (49.9)
70+	195 (19.3)
Missing	1
**Site**	
Right	429 (48.6)
Left	447 (50.7)
Bilateral	6 (0.7)
Missing	127
**Histologic type**	
IBC-NST	745 (78.1)
Lobular	62 (6.5)
Mucinous	2 (0.2)
Medullary	39 (4.1)
Mixed IBC-NST and invasive lobular	7 (0.7)
Apocrine	43 (4.5)
Papillary	7 (0.7)
Adenoid cystic	6 (0.6)
Metaplastic	42 (4.4)
Micropapillary	2 (0.2)
Missing	54
**Histologic grade**	
G1	11 (1.1)
G2	204 (21.3)
G3	744 (77.6)
Missing	50
**Tumor size**	
pT0	1 (0.1)
pT1	379 (40.6)
pT2	441 (47.3)
pT3	59 (6.3)
pT4	53 (5.7)
Missing	76
**Lymph node status**	
pN0	533 (59.4)
pN1	214 (23.9)
pN2	90 (10.0)
pN3	60 (6.7)
Missing	112
**Metastasis**	
M0	812 (95.8))
M1	36 (4.2
Missing	161
**TNM stage**	
I	258 (29.1)
II	413 (46.5)
III	182 (20.5)
IV	35 (3.9)
Missing	121
**Lymph node ratio**	
< 0,2	717 (81.4)
0,21-0,65	103 (11.7)
> 0,65	61 (6.9)
Missing	128
**Lymphovascular invasion**	
yes	349 (45.0)
no	427 (55.0)
Missing	233
**Necrosis**	
Present	491 (61.0)
Absent	314 (39.0)
Missing	184
**Lymphocytic infiltrate**	
yes	504 (64.5)
no	278 (35.5)
Missing	227
**Proliferation index (Ki-67)**	
< 14%	94 (9.9)
15–30%	155 (16.4)
≥ 30%	698 (73.7)
Missing	62
**Androgen Receptor**	
Positive	125 (24.3)
Negative	389 (75.7)
Missing	495
**Type of surgery**	
Mastectomy	341 (42.2)
Quadrantectomy	451 (55.5)
Lumpectomy	17 (2.0)
Missing	200
**Adjuvant chemotherapy**	
yes	580 (57.5)
no	429 (42.5)
**Adjuvant radiotherapy**	
yes	364 (36.1)
no	645 (63.9)
**Recurrence**	
yes	67 (6.6)
no	942 (93.4)
**Contralateral breast cancer**	
yes	69 (6.8)
no	940 (93.2)

*n* = number

Tables 2 and 3 outline the anamnestic data of TNBC patients and tumors characteristics according to histologic type. Patients affected by adenoid cystic (16.7%) or medullary (15.4%) carcinomas were frequently younger in age (< 35 years), whereas apocrine carcinoma (41.9%) was more frequent at > 70 years. The adenoid cystic tumors showed lower grade (G1) and low proliferation index (Ki67 < 14.0%), accounting for 33.3 and 33.3% of samples, respectively. Metaplastic and medullary types showed higher grades (G3), namely 91.1 and 89.2%, respectively, as well as higher proliferation index (Ki67: > 30.0%), with 84.4 and 89.5%, respectively. Lymph node ratio < 0.2 was reported in adenoid cystic (100.0%), metaplastic (86.4%) and medullary (91.4%) types; the lobular type showed the higher lymph node ratio (> 0.65), accounting for 20.4%. LVI was more frequent in lobular (51.6%) and apocrine carcinomas (50.0%); LVI was also detected in 33.3% of adenoid cystic and only in 7.4% of medullary carcinomas. No necrosis, TILs and AR expression were found in adenoid cystic carcinomas; the higher percentage of necrosis was identified in metaplastic carcinoma (83.7%), whereas the highest proportion of TILs was seen in medullary tumors (92.3%). As expected, lobular and apocrine types showed higher expression of AR than other histologic types.

**Table 2:** Anamnestic data of 1009 patients with “*Triple Negative”* breast cancer based on histologic subtypes. Sardinia, Italy 1994–2015

	IBC-NST (*n* = 744) n (%)	Lobular (*n* = 62) n (%)	Apocrine (*n* = 43) n (%)	Adenoid cystic (*n* = 6) n (%)	Metaplastic (*n* = 46) n (%)	Medullary (*n* = 39) n (%)	Other (*n* = 27) n (%)	*p* value*
**Age**								
< 35	34 (4.6)	0 (0.0)	2 (4.7)	1 (16.7)	3 (6.5)	6 (15.4)	1 (3.7)	**< 0.001**
35–49	214 (28.8)	10 (16.1)	1 (2.3)	1 (16.7)	8 (17.4)	12 (30.8)	8 (29.6)	
50–69	354 (47.6)	36 (58.1)	22 (51.2)	3 (50.0)	26 (56.5)	18 (46.2)	16 (59.3)	
70+	141 (19.0)	16 (25.8)	18 (41.9)	1 (16.7)	9 (19.6)	3 (7.7)	2 (7.4)	
Missing	1							
**Age at menarche**								
< 13	208 (45.9)	10 (31.3)	11 (37.9)	0 (0.0)	9 (27.3)	7 (43.8)	4 (19.0)	**0.036**
≥ 13	245 (54.1)	22 (68.8)	18 (62.1)	2 (100.0)	24 (72.7)	9 (56.3)	17 (81.0)	
Missing	291	30	14	4				
**Age at menopause**								
< 45	34 (11.8)	3 (12.0)	2 (7.7)	1 (50.0)	1 (4.3)	2 (18.2)	1 (7.7)	0.519
≥ 45	253 (88.2)	22 (88.0)	24 (92.3)	1 (50.0)	22 (95.7)	9 (81.8)	12 (92.3)	
Missing	457	37	17	3	23	27	14	
**Menopausal status**								
yes	210 (47.6)	18 (66.7)	17 (63.0)	1 (33.3)	20 (60.6)	10 (52.6)	12 (54.5)	0.124
post surgical	39 (8.8)	3 (11.1)	4 (14.8)	1 (33.3)	2 (6.1)	2 (10.5)	1 (4.5)	
post treatment	2 (0.5)	0 (0.0)	1 (3.7)	0 (0.0)	0 (0.0)	1 (5.3)	0 (0.0)	
no	190 (43.1)	6 (22.2)	5 (18.5)	1 (33.3)	11 (33.3)	6 (31.6)	9 (40.9)	
Missing	303	35	16	3	13	20	5	
**Nulliparity**								
yes	80 (16.4)	1 (3.1)	7 (25.0)	0 (0.0)	7 (19.4)	4 (25.0)	4 (20.0)	0.280
no	409 (83.6)	31 (96.9)	21 (75.0)	3 (100.0)	29 (80.6)	12 (75.0)	16 (80.0)	
Missing	255	30	15	3	10	22	7	
**Family history of breast cancer**								
yes	166 (33.8)	9 (23.7)	11 (36.7)	3 (100.0)	13 (37.1)	9 (45.0)	5 (21.7)	0.104
no	325 (66.2)	29 (76.3)	19 (63.3)	0 (0.0)	22 (62.9)	11 (55.0)	18 (78.3)	
Missing	253	24	13	3	11	19	4	
**Family history of other than breast cancer**								
yes	137 (29.1)	14 (38.9)	6 (20.7)	0 (0.0)	8 (25.0)	6 (35.3)	10 (45.5)	0.309
no	333 (70.9)	22 (61.1)	23 (79.3)	3 (100.0)	24 (75.0)	11 (64.7)	12 (54.5)	
Missing	274	26	14	3	14	20	5	
**Other concomitant primary cancer**								
yes	11 (4.5)	1 (5.0)	0 (0.0)	0 (0.0)	0 (0.0)	0 (0.0)	0 (0.0)	0.870
no	231 (95.5)	19 (95.0)	12 (100.0)	2 (100.0)	19 (100.0)	8 (100.0)	11 (100.0)	
Missing	502	42	31	4	27	31	16	
**Other previous primary cancer**								
yes	19 (7.3)	1 (5.0)	3 (20.0)	0 (0.0)	0 (0.0)	1 (11.1)	0 (0.0)	0.361
no	242 (92.7)	19 (95.0)	12 (80.0)	2 (100.0)	19 (100.0)	8 (88.9)	11 (100.0)	
Missing	483	42	28	4	27	30	16	
**BMI**								
< 18 underweight	14 (3.0)	0 (0.0)	0 (0.0)	1 (33.3)	0 (0.0)	0 (0.0)	2 (10.0)	0.183
18–24.9 normal range	235 (50.8)	15 (46.9)	11 (42.3)	1 (33.3)	18 (54.5)	8 (47.1)	8 (40.0)	
25–30 overweight	135 (29.2)	11 (34.4)	7 (26.9)	1 (33.3)	11 (33.3)	7 (41.2)	7 (35.0)	
> 30 0bese	79 (17.1)	6 (18.8)	8 (30.8)	0 (0.0)	4 (12.1)	2 (11.8)	3 (15.0)	
Missing	281	30	17	3	13	22	7	
**Drinking**								
yes	21 (4.9)	1 (3.1)	0 (0.0)	1 (33.3)	0 (0.0)	3 (21.4)	1 (4.8)	**0.015**
no	407 (95.1)	31 (96.9)	22 (100.0)	2 (66.7)	32 (100.0)	11 (78.6)	20 (95.2)	
Missing	316	30	21	3	14	24	6	
**Smoking**								
yes	92 (20.2)	5 (14.7)	4 (16.0)	1 (3.3)	7 (21.2)	3 (18.8)	3 (13.6)	0.942
no	364 (79.8)	29 (85.3)	21 (84.0)	2 (66.7)	26 (78.8)	13 (81.3)	19 (86.4)	
Missing	288	28	18	3	13	22	5	

*The *p*-value are bold where they are less than or equal to the significance level of 0.05

**Table 3:** Clinico-pathological data of 1009 patients with “*Triple Negative”* breast cancer based on histologic subtypes. Sardinia, Italy 1994–2015

	IBC-NST (*n* = 744) n (%)	Lobular (*n* = 62) n (%)	Apocrine (*n* = 43) n (%)	Adenoid cystic (*n* = 6) n (%)	Metaplastic (*n* = 46) (%)	Medullary (*n* = 39) n (%)	Other (*n* = 27) n (%)	*p* value*
**Site**								
Right	328 (49.1)	23 (52.2)	15 (44.1)	3 (50.0)	16 (38.1)	17 (48.6)	12 (50.0)	0.977
Left	334 (50.0)	21 (47.7)	19 (55.9)	3 (50.0)	26 (61.9)	18 (51.4)	12 (50.0)	
Bilateral	6 (0.9)	0 (0.0)	0 (0.0)	0 (0.0)	0 (0.0)	0 (0.0)	0 (0.0)	
Missing	76	18	9		4	4	3	
**Histologic grade**								
G1	5 (0.7)	1 (2.0)	2 (4.7)	2 (33.3)	0 (0.0)	1 (2.7)	1 (4.0)	**< 0.001**
G2	128 (17.8)	25 (50.0)	18 (41.9)	3 (50.0)	4 (8.9)	3 (8.1)	8 (32.0)	
G3	588 (81.6)	24 (48.0)	23 (53.5)	1 (16.7)	41 (91.1)	33 (89.2)	16 (64.0)	
Missing	23	12			1	2	2	
**Tumor size**								
pT0	1 (0.1)	0 (0.0)	0 (0.0)	0 (0.0)	0 (0.0.)	0 (0.0)	0 (0.0)	0.110
pT1	285 (39.7)	14 (24.6)	22 (51.2)	5 (83.3)	13 (28.3)	17 (44.7)	8 (32.0)	
pT2	337 (47.0)	32 (56.1)	17 (39.5)	1 (16.7)	22 (47.8)	17 (44.7)	9 (36.0)	
pT3	32 (4.5)	7 (12.3)	3 (7.0)	0 (0.0)	9 (19.6)	3 (7.9)	4 (16.0)	
pT4	43 (6.0)	2 (3.5)	1 (2.3)	0 (0.0)	2 (4.3)	1 (2.6)	3 (12.0)	
pTx	18 (2.5)	2 (3.5)	0 (0.0)	0 (0.0)	0 (0.0)	0 (0.0)	1 (4.0)	
Tis	1 (0.1)	0 (0.0)	0 (0.0)	0 (0.0)	0 (0.0)	0 (0.0)	0 (0.0)	
Missing	27	5				1	2	
**Lymph node status**								
pN0	395 (56.3)	22 (38.6)	28 (65.1)	3 (60.0)	30 (65.2)	24 (63.2)	10 (40.0)	0.190
pN1	155 (22.1)	15 (26.3)	7 (16.3)	2 (40.0)	11 (23.9)	11 (28.9)	11 (44.0)	
pN2	68 (9.7)	9 (15.8)	5 (11.6)	0 (0.0)	4 (8.7)	1 (2.6)	2 (8.0)	
pN3	47 (6.7)	7 (12.3)	3 (7.0)	0 (0.0)	0 (0.0)	1 (2.6)	1 (4.0)	
pNx	36 (5.1)	4 (7.0)	0 (0.0)	0 (0.0)	1 (2.2)	1 (2.6)	1 (4.0)	
Missing	43	5		1		1	2	
**Metastasis**								
M0	649 (87.2)	55 (88.7)	41 (95.3)	5 (83.3)	41 (89.1)	38 (97.4)	21 (77.8)	0.190
M1	95 (12.8)	7 (11.3)	2 (4.7)	1 (16.7)	5 (10.9)	1 (2.6)	6 (22.2)	
**TNM stage**								
I	188 (28.5)	8 (15.1)	19 (44.2)	3 (50.0)	10 (22.2)	11 (29.7)	5 (21.7)	0.210
II	306 (46.4)	27 (50.9)	15 (34.9)	2 (33.3)	24 (53.3)	23 (62.2)	10 (43.5)	
III	137 (20.8)	15 (28.3)	9 (20.9)	1 (16.7)	9 (20.0)	2 (5.4)	7 (30.4)	
IV	28 (4.2)	3 (5.7)	0 (0.0)	0 (0.0)	2 (4.4)	1 (2.7)	1 (4.3)	
Missing	85	9			1	2	4	
**Lympho node ratio**								
< 0.2	520 (81.0)	35 (64.8)	36 (85.7)	5 (100.0)	38 (86.4)	32 (91.4)	19 (82.6)	**0.019**
0.21–0.65	75 (11.7)	8 (14.8)	4 (9.5)	0 (0.0)	5 (11.4)	3 (8.6)	4 (17.4)	
> 0.65	47 (7.3)	11 (20.4)	2 (4.8)	0 (0.0)	1 (2.3)	0 (0.0)	0 (0.0)	
Missing	102	8	1	1	2	4	4	
**Proliferation index (Ki-67)**								
< 14%	54 (7.6)	20 (37.0)	7 (16.3)	2 (33.3)	2 (4.4)	2 (5.3)	3 (11.1)	**< 0.001**
15–30%	110 (15.6)	11 (20.4)	13 (30.2)	1 (16.7)	5 (11.1)	2 (5.3)	8 (29.6)	
≥ 30%	542 (76.8)	23 (42.6)	23 (53.5)	3 (50.0)	38 (84.4)	34 (89.5)	16 (59.3)	
Missing	38	8			1	1		
**In situ component**								
Present	112 (15.1)	2 (3.2)	8 (18.6)	0 (0.0)	3 (6.5)	2 (5.1)	4 (14.8)	**0.038**
Absent	632 (84.9)	60 (96.8)	35 (81.4)	6 (100.0)	43 (93.5)	37 (94.9)	23 (85.2)	
**Lymphovascular invasion**								
yes	276 (46.0)	16 (51.6)	18 (50.0)	2 (33.3)	18 (42.9)	2 (7.4)	12 (60.0)	**0.004**
no	324 (54.0)	15 (48.4)	18 (50.0)	4 (66.7)	24 (57.1)	25 (92.6)	8 (40.0)	
Missing	144	31	7		4	12	7	
**Necrosis**								
Present	388 (62.7)	13 (37.1)	15 (39.5)	0 (0.0)	36 (83.7)	13 (50.0)	12 (60.0)	**< 0.001**
Absent	231 (37.3)	22 (62.9)	23 (60.5)	6 (100.0)	7 (16.3)	13 (50.0)	8 (40.0)	
Missing	125	27	5		3	13	7	
**Lymphocytic infiltrate**								
yes	397 (65.2)	11 (36.7)	27 (75.0)	0 (0.0)	24 (60.0)	24 (92.3)	13 (65.0)	**< 0.001**
no	212 (34.8)	19 (63.3)	9 (25.0)	6 (100.0)	16 (40.0)	2 (7.7)	7 (35.0)	
Missing	135	32	7		6	13	7	
**Androgen receptor**								
Positive	79 (19.6)	10 (76.9)	24 (88.9)	0 (0.0)	3 (9.7)	1 (6.3)	4 (33.3)	**< 0.001**
Negative	324 (80.4)	3 (23.1)	3 (11.1)	4 (100.0)	28 (90.3)	15 (93.8)	8 (66.7)	
Missing	341	49	16	2	15	23	15	
**Type of surgery**								
Mastectomy	244 (40.7)	23 (53.5)	19 (51.4)	0 (0.0)	19 (59.4)	12 (35.3)	12 (50.0)	0.369
Quadrantectomy	342 (57.0)	20 (46.5)	18 (48.6)	4 (100.0)	21 (65.6)	22 (64.7)	11 (45.8)	
Lumpectomy	14 (2.3)	0 (0.0)	0 (0.0)	0 (0.0)	2 (6.3)	0 (0.0)	1 (4.2)	
Missing	144	19	6	2	4	5	3	
**Adjuvant chemotherapy**								
yes	436 (58.6)	34 (54.8)	25 (58.1)	2 (33.3)	29 (63.0)	22 (56.4)	19 (70.4)	0.685
no	308 (41.4)	28 (45.2)	18 (41.9)	4 (66.7)	17 (37.0)	17 (43.6)	8 (29.6)	
**Adjuvant radiotherapy**								
**yes**	258 (34.7)	17 (27.4)	20 (46.5)	1 (16.7)	19 (41.3)	14 (35.9)	12 (44.4)	**< 0.001**
no	468 (65.3)	45 (72.6)	23 (53.5)	5 (83.3)	27 (58.7)	25 (64.1)	15 (55.6)	

*The *p*-value are bold where they are less than or equal to the significance level of 0.05

Supplementary Figure 1 (see Additional file 1) shows the distribution of tumor size according to TNBC histologic type: using IBC-NST as a reference the adenoid cystic carcinoma displayed the smallest size (median size: 14.5 mm vs 21 mm for IBC-NST; *p* = 0.050), with the distributions of tumor size relatively concentrated [Interquartile range (IQR): 13.2–18.7 mm]. Although, apocrine, lobular and metaplastic carcinomas showed higher tumor size than IBC-NST, the *p* value did not achieve significance. A significant positive correlation between tumor size and number of metastatic lymph nodes was identified in apocrine and metaplastic carcinomas, with a Spearman correlation coefficient of 0.507, *p* = 0.031 and 0.384, *p* = 0.039, respectively (see Additional file 1: Supplementary Table 1). Linear regression model used to analyze the effect of tumor size on the number of metastatic lymph nodes, showing that the slope of the regression line for apocrine carcinoma (slope 0.171 vs 0.006; *p* = 0.040) was significantly higher than IBC-NST (see Additional file 1: Supplementary Figure 2).

### Prognostic indicators according to TNBC histologic types

**Fig. 1:**
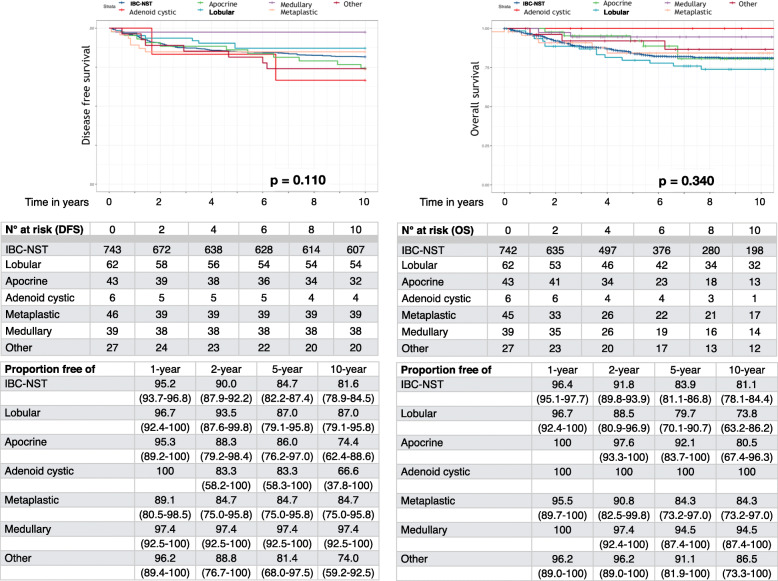
Kaplan-Meier curves of disease free survival (**a**) and overall survival (**b**) of patients affected by “*Triple Negative*” breast cancer according to histologic types

The Kaplan-Meier curves for DFS and OS according to TNBC histologic type did not show statistically significance with *p* = 0.110 and *p* = 0.340, respectively (Fig. 1). Although patients with adenoid cystic carcinoma had the best 1-year DFS (100.0%); conversely, patients with metaplastic carcinoma showed the worst DFS (89.1%). At five-years, DFS was similar for patients with lobular (87.0%), apocrine (86.0%), adenoid cystic (83.3%) and metaplastic (84.7%) carcinomas, while the patients affected by medullary carcinoma showed the best DFS (97.4%). At ten-years, patients with medullary carcinoma still showed the most favorable behavior (97.4% of cases), while patients with adenoid cystic carcinoma showed the highest rate of relapse (33.4%) among all types.

Moreover, at 1-year follow-up OS was 100.0% for patients with adenoid cystic, apocrine and medullary carcinoma. At five-years, OS was 92.1, 100.0, and 94.5% for patients with apocrine, adenoid cystic and medullary carcinoma, respectively; conversely, patients affected by lobular and metaplastic carcinoma showed the worst OS, 79.7 and 84.3%, respectively. Finally, at ten-years, patients with adenoid cystic (100.0%) and medullary (94.5%) carcinoma had a more favorable prognosis, while patients with lobular carcinoma showed the worst prognosis (73.8%).

The multivariate analysis revealed that TNBC medullary type showed an independent prognostic factor for DFS compared to IBC-NST (HR, 0.12; 95% CI 0.01–0.87; *p* = 0.030). The same pattern was observed for OS, even though it was not statistically significant (Table 4).

**Table 4:** Hazard ratios (HRs) of disease-free survival and mortality, and corresponding 95% of confidence intervals (CIs), according to histologic subtypes, among 1009 “*Triple Negative*” breast cancer. Sardinia, Italy 1994–2015

	Disease-free survival		Overall survival	
	Univariate analysis HR (95% CI)	Multivariate analysis^**a**^ HR (95% CI)	Univariate analysis HR (95% CI)	Multivariate analysis^**a**^ HR (95% CI)
IBC-NST	1.00^b^	1.00^b^	1.00^b^	1.00^b^
Lobular	0.68 (0.33–1.39)	0.57 (0.26–1.24)	1.36 (0.79–2.33)	1.06 (0.50–2.25)
Apocrine	1.40 (0.76–2.60)	1.34 (0.71–2.49)	0.77 (0.64–1.76)	0.54 (0.17–1.73)
Adenoid cystic	1.85 (0.46–7.50)	1.20 (0.16–8.69)	–	–
Metaplastic	0.85 (0.40–1.83)	0.75 (0.34–1.64)	0.88 (0.39–2.01)	0.60 (0.18–1.96)
Medullary	**0.13 (0.01–0.92)**	**0.12 (0.01–0.87)**	0.30 (0.07–1.21)	0.42 (0.10–1.74)
Other	1.44 (0.67–3.09)	1.33 (0.61–2.89)	0.64 (0.20–2.04)	0.17 (0.02–1.28)

^a^Estimates from multivariate proportional hazard regression model adjusted for age, tumor size and number of positive nodes. Estimates in bold are those significant at the 0.05 level

^b^Reference category

## Discussion

The present study investigated the clinico-pathological features and prognosis of different TNBC histologic types. The incidence of TNBC histologic variants analyzed was concordant with other studies [11, 14, 22].

Taking IBC-NST as a reference, our data showed that patients with lobular and metaplastic carcinoma had poor survival outcomes at 5- and 10-years follow-up; patients with adenoid cystic and medullary carcinoma had excellent prognosis at 5- and 10-years follow-up, and patients with apocrine carcinoma had good outcome until 5-years, with similar values to IBC-NST at 10-years. Patients with medullary carcinoma had the best DFS: based on the multivariate analysis medullary TNBC is an independent prognostic factor for DFS. Specific clinico-pathological and molecular features could explain the dissimilarities in the outcome of patients affected by TNBC with different histologic types.

Our results revealed that patients affected by invasive lobular carcinoma with triple negative phenotype had a poorer prognosis (79.7% at 5-years and 73.8% at 10-years) compared to all other histologic types. Triple negative invasive lobular carcinomas were mainly solid or mixed classic and solid variants, with only 3 cases showing morphologic features of pleomorphic variants. Our results showed that invasive lobular carcinoma represented a TNBC morphologic variant with higher lymph node ratio (> 0.65), higher AR expression and lower TILs component, than those of other histologic special types. Moreover, 63% of triple negative invasive lobular carcinoma had a proliferation index (Ki-67) > 15.0%.

Previous studies showed conflicting findings on the prognosis of lobular carcinoma patients compared to IBC-NST [23, 24], although prevalent data were obtained by analyzing ER-positive invasive lobular carcinoma and IBC-NST. Our results are supported by other authors’ findings, especially when ER-negative invasive lobular carcinoma and IBC-NST were compared [14, 25–28].

Recently, molecular analyses of invasive lobular carcinomas established that these tumors have a distinct genomic profile compared to IBC-NST, exhibiting a high frequency of *CDH1* mutations, loss of *PTEN*, activation of *AKT*, and mutations in *TBX3* and *FOXA1* [29, 30]. In addition, different studies found that patients with IBC-NST derive greater benefit from chemotherapy than patients diagnosed with invasive lobular carcinoma [31–35].

In the present study, metaplastic carcinoma resulted as a TNBC morphologic variant with poor prognosis, as previously described in the literature [12–14]. The 46 metaplastic carcinomas analyzed in this study exhibited high grade (91.1%), high Ki67 expression (84.4%), and presence of necrosis (83.7%), which might explain their poor prognosis. Interestingly, metaplastic breast cancers were found to express genes involved in epithelial mesenchymal transition and cell motility pathways, as observed in TNBC mesenchymal-like molecular subtype [36, 37]. The biological behavior of metaplastic carcinomas might be related to *β-Catenin* gene mutations and activation of WNT pathway [36, 38]. Furthermore, metaplastic tumors were included in mesenchymal-like molecular subtype based on their gene expression profile, characterized by PI3K/mTOR pathway aberrations, downregulation of genes involved in DNA repair and response to chemotherapy. These genetic deregulations could be responsible for the resistance to conventional chemotherapy showed by this histologic subtype [39], in contrast with the general chemosensitivity of IBC-NST with Triple Negative phenotype.

In the current study, patients with medullary, apocrine and adenoid cystic carcinomas had better prognosis compared to IBC-NST. The classic medullary carcinomas analyzed in this study showed significantly high lymphocytic infiltrate (92.3%) and no lymphovascular invasion (92.6%), with an overall survival of 94.5% at 10-years follow-up, regardless of high grade and proliferation index. Recent studies demonstrated that immune signaling genes within the immunomodulatory molecular TNBC subtype substantially overlap with gene signature of medullary breast cancer [36–40]. The absence of lymphovascular invasion might explain the good prognosis of medullary carcinoma, which might be related to downregulation of genes associated with cell invasiveness [40, 41].

Furthermore, Denkert et al. described a quantitative assessment of TILs as a predictor of response to neoadjuvant chemotherapy [42]. Retrospective analysis of clinical trials confirmed that TILs levels are predictive of pathologic complete response and increased disease-free and overall survival [20, 43].

In addition, recent evidence has shown the prognostic importance of high TILs in high grade BC such as medullary carcinoma, usually associated with good prognosis. Recently, the new WHO classification of Breast Tumors has included carcinoma with medullary features in the spectrum of TILs-rich IBC-NST, defined as IBC-NST with medullary pattern, and no longer as a distinct special subtype [44]. Leon-Ferre et al. evaluated the prognostic role of TILs in TNBC patients who did not receive adjuvant chemotherapy, demonstrating that TILs is an independent prognostic factor in early-stage TNBC. They speculated that high TILs may represent the activation of an endogenous antitumor immune response that occurs even in the absence of immune enhancements triggered by chemotherapy. Specifically, univariate analysis showed that carcinomas with medullary features have better outcomes when compared to invasive carcinomas of NST, whereas this association was lost once TILs were included into a multivariate model [45].

Recently, a higher immunohistochemical expression of PD-L1 known to be associated with immune evasion in a variety of malignancies, including TNBC, was identified in breast carcinomas with medullary features, in which might represent a marker of susceptibility to PD-1/PD-L1 inhibitor therapies [46].

Our data showed that patients affected by apocrine carcinoma had better overall survival compared to IBC-NST at 5-years follow-up, while the OS was similar for the two histologic types at 10-years, showing concordance with results of Takeuchi et al. [47]. Apocrine carcinomas were intermediate/high grade morphologic variants, with low lymph node ratio and high lymphocytic infiltrate and AR expression, and diagnosed at older age (41.9% at > 70 years). AR signaling pathway activation is a prominent feature for apocrine lesions of the breast, representing a therapeutic opportunity by androgen therapies [48]. So far, immunotherapies based on immune checkpoint inhibitors have not been specifically proposed for apocrine carcinomas, due to the low level or absent expression of PD-L1, in contrast to BC with triple negative phenotype, which is known to be variably PD-L1 positive (9–59%) [46, 49]. Our data highlighted a higher percentage of apocrine carcinomas with lymphocytic infiltrate (75.0%) than the percentage showed in IBC-NST (65.2%), suggesting that immunotherapies based on immune checkpoint inhibitors might be taken into consideration also for this TNBC special type.

Adenoid cystic carcinoma is a rare variant of breast cancer, accounting for less than 1% of all invasive breast cancers, which shows basal-like features and triple negative phenotype, but with a favorable clinical course. Although in our *TNBC Database* only six adenoid cystic carcinomas were available, namely 5 classic type and 1 solid-basaloid type, they were analyzed as a group according to its specificity and in accordance with other studies [50]. Patients with adenoid cystic carcinoma had an OS of 100% at 10-years follow-up, with prevalently low/intermediate grade, low lymph node ratio, absence of necrosis, lymphocytic infiltrate, and AR expression. Uncommonly, our results showed that patients with adenoid cystic carcinoma had the highest rate of relapse (33.4%) among all histologic types. These results are conflicting and need a thorough analysis on a large casuistry. Furthermore, five case reports on patients with breast adenoid cystic carcinoma showing one or more distant metastasis have been published [51]. Chen et al. using a large number of population-base data showed that breast adenoid cystic carcinoma has different pathological features and good clinical course compared to IBC-NST, but comparing BC *Triple Negative* phenotype no differences were shown between the two histologic types as far as recurrence and mortality are concerned [52]. Patients with adenoid cystic carcinoma exhibited smaller size and limited distribution of tumor size than patients with IBC-NST (*p* = 0.050), according to the results of Mills et al. [12]. Conversely, *Chen* et al. did not report any differences between tumor size in adenoid cystic carcinoma compared to IBC-NST [47], while Kulkarn et al. found that patients with adenoid cystic carcinoma had larger median tumor size than patients with IBC-NST [53].

Genetically, *MYB-NFIB* fusion gene is a prevalent feature of adenoid cystic carcinoma. Recently, a whole exome sequencing performed on 12 adenoid cystic carcinomas demonstrated that no somatic mutations in *TP53*, *PIK3CA*, *RB1*, *BRCA1* or *BRCA2* genes were identified in breast adenoid cystic carcinomas, unlike what occurs in common-type triple-negative and basal-like breast cancers. Interestingly, the mutational status of breast adenoid cystic carcinomas is more similar to those of salivary gland adenoid cystic carcinoma, than in other TNBCs types, emphasizing the importance of TNBCs histologic subtyping [54].

Considering tumor size and lymph nodes status as significant clinico-pathological features in the choice of therapeutic regimens and in the prognosis of TNBC histologic types, the correlation between these parameters was analyzed. Tumor size and number of metastatic lymph nodes showed a positive correlation in apocrine and metaplastic carcinomas. Interestingly, by using a linear regression model it was observed that the increase of tumor size correlates with a great increase of average number of metastatic lymph nodes in apocrine carcinoma compared to IBC-NST. These data differ from Zhao et al. results, who demonstrated a positive correlation between tumor size and number of metastatic lymph nodes in different special types, such as lobular carcinoma and mixed ductal (NST)-lobular variants [13].

Our study strengthens previous clinical and experimental data in favoring greater integration between histologic and molecular characterization of these variants, which should be considered together in the choice of therapeutic regimens.

Current clinical data indicate that patients with triple negative lobular or metaplastic carcinoma are less responsive to chemotherapy, contrary to patients affected by other TNBC histologic types, increasing the need to identify unambiguous molecular targets. Conversely, patients with TNBC histologic variants as medullary, apocrine and adenoid cystic carcinomas, both characterized by a more favorable prognosis, could be treated with less aggressive or even without chemotherapeutic regimens, according to the disease stage. Moreover, new therapeutic approaches, such as immunotherapy based on immune checkpoint inhibitors, or androgen-targeting therapies, should be evaluated in the treatment of TNBC patients according to histologic “*special types*”.

The main limitation of this study is represented by inherent biases dependent on the retrospective nature of the design, notwithstanding three pathologists according to current WHO classification [16] achieved definitive histologic subtyping of all the cases. Moreover, ER, PgR and HER2 immunohistochemical results for TNBC samples according to the ASCO/CAP recommendations were standardized [17]. Unfortunately, for some patients important clinical and pathological data were missing because not originally included in the medical records, and those missing information might have influenced to some extent the evaluated associations.

## Conclusions

The main conclusion of the present study is that clinico-pathological features and prognosis of TNBC differ according to histologic types. Adenoid cystic carcinoma, apocrine carcinoma and IBC-NST with medullary pattern, have in common a favourable prognosis, while invasive lobular carcinoma and metaplastic carcinoma are the most aggressive subtypes.

Our study confirms that an accurate and reliable histopathologic definition of TNBC subtypes has a significant clinical utility and is an effective tool during the therapeutic decision-making process, with the aim to develop innovative and personalized treatments.

## Supplementary information

**Additional file 1: Figure S1.** Violin plot of tumor size distribution according to “Triple Negative” breast cancer histologic types. **Table S1.** Correlation between tumor size and number of metastatic lymph nodes in different histologic types of “Triple Negative” breast cancer. Sardinia, Italy 1994–2015. **Figure S2.** Correlation between tumor size and number of positive lymph nodes in “Triple Negative” breast cancer histologic types. A linear regression model constructs the regression lines. The slope of the regression line symbolizes the average increase in the number of metastatic lymph nodes for each millimeter increase in tumor size for each histologic TNBC types, indicative of the influence of tumor size on number of metastatic lymph nodes.

## Data Availability

The dataset analyzed during the current study is available from the corresponding author on reasonable request.

## Abbreviations

TNBC: Triple Negative breast cancer
IBC-NST: Invasive breast carcinomas of no special type
OS: Overall survival
DFS: Disease free survival
BC: Breast cancer
ER: Estrogen receptor
PR: Progesterone receptor
IHC: Immunohistochemistry
SISH: In situ hybridization
AR: Androgen receptors
ASCO/CAP: American Society of Clinical Oncology/College of American Pathologists recommendations
LVI: Lymphovascular invasion
TILs: Tumor-infiltrating lymphocytes
HR: Hazard ratio
CI: Confidence intervals
IQR: Interquartile range

## References

[1] Perez EA (2011). Breast cancer management: opportunities and barriers to an individualized approach. Oncologist.

[2] Perou CM, Sørlie T, Eisen MB, van de Rijn M, Jeffrey SS, Rees CA (2000). Molecular portraits of human breast tumours. Nature..

[3] De Abreu FB, Wells WA, Tsongalis GJ (2013). The emerging role of the molecular diagnostics laboratory in breast cancer personalized medicine. Am J Pathol.

[4] Stingl J, Caldas C (2007). Molecular heterogeneity of breast carcinomas and the cancer stem cell hypothesis. Nat Rev Cancer.

[5] Bastien RRL, Rodriguez-Lescure A, Ebbert MTW, Prat A, Munárriz B, Rowe L (2012). PAM50 breast cancer subtyping by RT-qPCR and concordance with standard clinical molecular markers. BMC Med Genet.

[6] Carey LA, Perou CM, Livasy CA, Dressler LG, Cowan D, Conway K (2006). Race, breast cancer subtypes, and survival in the Carolina Breast Cancer Study. J Am Med Assoc.

[7] Metzger-Filho O, Tutt A, de Azambuja E, Saini KS, Viale G, Loi S (2012). Dissecting the heterogeneity of triple-negative breast cancer. J Clin Oncol.

[8] Reis-Filho JS, Tutt ANJ (2008). Triple negative tumours: a critical review. Histopathology..

[9] Rakha EA, Allison KH, Bu H, Cree IA, Lokuhetty D (2019). Invasive breast carcinoma of no special type. World Health Organization classification of the tumors-breast tumors.

[10] Page DL (2003). Special types of invasive breast cancer, with clinical implications. Am J Surg Pathol.

[11] Montagna E, Maisonneuve P, Rotmensz N, Cancello G, Iorfida M, Balduzzi A (2013). Heterogeneity of triple-negative breast cancer: histologic subtyping to inform the outcome. Clin Breast Cancer.

[12] Mills MN, Yang GQ, Oliver DE, Liveringhouse CL, Ahmed KA, Orman AG (2018). Histologic heterogeneity of triple negative breast cancer: a National Cancer Centre Database analysis. Eur J Cancer.

[13] Zhao S, Ma D, Xiao Y, Jiang YZ, Shao ZM (2018). Clinicopathologic features and prognoses of different histologic types of triple-negative breast cancer: a large population-based analysis. Eur J Surg Oncol.

[14] Liao HY, Zhang WW, Sun JY, Li FY, He ZY, Wu SG (2017). The clinicopathological features and survival outcomes of different histological subtypes in triple-negative breast cancer. J Cancer.

[15] Urru SAM, Gallus S, Bosetti C, Moi T, Medda R, Sollai E (2018). Clinical and pathological factors influencing survival in a large cohort of triple-negative breast cancer patients. BMC Cancer.

[16] Rakha EA, Allison KH, Ellis IO, Horii R, Masuda S, Penault-Llorca F, Cree IA, Lokuhetty D (2019). Invasive breast carcinoma: general overview. World Health Organization classification of the tumors-breast tumors.

[17] Hammond ME, Hayes DF, Wolff AC, Mangu PB, Temin S (2010). American Society of Clinical Oncology/College of American Pathologists Guideline Recommendations for Immunohistochemical testing of estrogen and progesterone receptors in breast cancer. J Oncol Pract.

[18] Jongen L, Floris G, Wildiers H, Claessens F, Richard F, Laenen A (2019). Tumor characteristics and outcome by androgen receptor expression in triple-negative breast cancer patients treated with neo-adjuvant chemotherapy. Breast Cancer Res Treat.

[19] Denkert C, Wienert S, Poterie A, Loibl S, Budczies J, Badve S (2016). Standardized evaluation of tumor-infiltrating lymphocytes in breast cancer: results of the ring studies of the international immuno-oncology biomarker working group. Mod Pathol.

[20] Loi S, Sirtaine N, Piette F, Salgado R, Viale G, Van Eenoo F (2013). Prognostic and predictive value of tumor-infiltrating lymphocytes in a phase III randomized adjuvant breast cancer trial in node-positive breast cancer comparing the addition of Docetaxel to doxorubicin with doxorubicin-based chemotherapy: BIG 02-98. J Clin Oncol.

[21] Vinh-Hung V, Verkooijen HM, Fioretta G, Neyroud-Caspar I, Rapiti E, Vlastos G (2009). Lymph node ratio as an alternative to pN staging in node-positive breast cancer. J Clin Oncol.

[22] Dreyer G, Vandorpe T, Smeets A, Forceville K, Brouwers B, Neven P (2013). Triple negative breast cancer: clinical characteristics in the different histological subtypes. Breast..

[23] Chen Z, Yang J, Li S, Lv M, Shen Y, Wang B (2017). Invasive lobular carcinoma of the breast: A special histological type compared with invasive ductal carcinoma. PLoS One.

[24] Azim HA, Malek RA, Azim HA (2014). Pathological features and prognosis of lobular carcinoma in Egyptian breast cancer patients. Women Health.

[25] Lim ST, Yu JH, Park HK, Moon BI, Ko BK, Suh YJ (2014). A comparison of the clinical outcomes of patients with invasive lobular carcinoma and invasive ductal carcinoma of the breast according to molecular subtype in a Korean population. World J Surg Oncol.

[26] Iorfida M, Maiorano E, Orvieto E, Maisonneuve P, Bottiglieri L, Rotmensz N (2012). Invasive lobular breast cancer: subtypes and outcome. Breast Cancer Res Treat.

[27] Rakha EA, Ellis IO (2010). Lobular breast carcinoma and its variants. Semin Diagn Pathol.

[28] Barroso-Sousa R, Metzger-Filho O (2016). Differences between invasive lobular and invasive ductal carcinoma of the breast: results and therapeutic implications. Ther Adv Med Oncol.

[29] Ciriello G, Gatza ML, Beck AH, Wilkerson MD, Rhie SK, Pastore A (2015). Comprehensive molecular portraits of invasive lobular breast cancer. Cell..

[30] Desmedt C, Zoppoli G, Gundem G, Pruneri G, Larsimont D, Fornili M (2016). Genomic characterization of primary invasive lobular breast cancer. J Clin Oncol.

[31] Mathieu MC, Rouzier R, Llombart-Cussac A, Sideris L, Koscielny S, Travagli JP (2004). The poor responsiveness of infiltrating lobular breast carcinomas to neoadjuvant chemotherapy can be explained by their biological profile. Eur J Cancer.

[32] Cristofanilli M, Hayes DF, Budd GT, Ellis MJ, Stopeck A, Reuben JM (2005). Circulating tumor cells: a novel prognostic factor for newly diagnosed metastatic breast cancer. J Clin Oncol.

[33] Tubiana-Hulin M, Stevens D, Lasry S, Guinebretière JM, Bouita L, Cohen-Solal C (2006). Response to neoadjuvant chemotherapy in lobular and ductal breast carcinomas: a retrospective study on 860 patients from one institution. Ann Oncol.

[34] Lips EH, Mukhtar RA, Yau C, de Ronde JJ, Livasy C, Carey LA (2012). Lobular histology and response to neoadjuvant chemotherapy in invasive breast cancer. Breast Cancer Res Treat.

[35] Delpech Y, Coutant C, Hsu L, Barranger E, Iwamoto T, Barcenas CH (2013). Clinical benefit from neoadjuvant chemotherapy in oestrogen receptor-positive invasive ductal and lobular carcinomas. Br J Cancer.

[36] Lehmann BD, Bauer JA, Chen X, Sanders ME, Chakravarthy AB, Shyr Y (2011). Identification of human triple-negative breast cancer subtypes and preclinical models for selection of targeted therapies. J Clin Invest.

[37] Weigelt B, Kreike B, Reis-Filho JS (2009). Metaplastic breast carcinomas are basal-like breast cancers: a genomic profiling analysis. Breast Cancer Res Treat.

[38] Hayes MJ, Thomas D, Emmons A, Giordano TJ, Kleer CG (2008). Genetic changes of Wnt pathway genes are common events in metaplastic carcinomas of the breast. Clin Cancer Res.

[39] Moulder S, Moroney J, Helgason T, Wheler J, Booser D, Albarracin C (2011). Responses to liposomal doxorubicin, bevacizumab, and temsirolimus in metaplastic carcinoma of the breast: biologic rationale and implications for stem-cell research in breast cancer. J Clin Oncol.

[40] Bertucci F, Finetti P, Cervera N, Charafe-Jauffret E, Mamessier E, Adélaïde J (2006). Gene expression profiling shows medullary breast cancer is a subgroup of basal breast cancers. Cancer Res.

[41] Weigelt B, Horlings HM, Kreike B, Hayes MM, Hauptmann M, Wessels LF (2008). Refinement of breast cancer classification by molecular characterization of histological special types. J Pathol.

[42] Denkert C, Loibl S, Noske A, Roller M, Müller BM, Komor M (2010). Tumor-associated lymphocytes as an independent predictor of response to neoadjuvant chemotherapy in breast cancer. J Clin Oncol.

[43] Adams S, Gray RJ, Demaria S, Goldstein L, Perez EA, Shulman LN (2014). Prognostic value of tumor-infiltrating lymphocytes in triple-negative breast cancers from two phase III randomized adjuvant breast cancer trials: ECOG 2197 and ECOG 1199. J Clin Oncol.

[44] Rakha EA, Aleskandarany M, El-Sayed ME, Blamey RW, Elston RW, Ellis IO (2009). The prognostic significance of inflammation and medullary histological type in invasive carcinoma of the breast. Eur J Cancer.

[45] Leon-Ferre RA, Polley M-Y, Liu H, Gilbert JA, Cafourek V, Hillman DW (2018). Impact of histopathology, tumor-infiltrating lymphocytes, and adjuvant chemotherapy on prognosis of triple-negative breast cancer. Breast Cancer Res Treat.

[46] Dill EA, Gru AA, Atkins KA, Friedman LA, Moore ME, Bullock TN (2017). PD-L1 expression and intratumoral heterogeneity across breast cancer subtypes and stages: an assessment of 245 primary and 40 metastatic tumors. Am J Surg Pathol.

[47] Takeuchi H, Tsuji K, Ueo H, Kano T, Maehara Y (2004). Clinicopathological feature and long-term prognosis of apocrine carcinoma of the breast in Japanese women. Breast Cancer Res Treat.

[48] Mills AM, Gottlieb CE, Wendroth SM, Brenin CM, Atkins KA (2016). Pure apocrine carcinomas represent a Clinicopathologically distinct androgen receptor-positive subset of triple-negative breast cancers. Am J Surg Pathol.

[49] Vranic S, Feldman R, Gatalica Z (2017). Apocrine carcinoma of the breast: a brief update on the molecular features and targetable biomarkers. Bosn J Basic Med Sci.

[50] McClenathan JH, de la Roza G (2002). Adenoid cystic breast cancer. Am J Surg.

[51] Mhamdi HA, Kourie HR, Jungels C, Aftimos F, Belbaraka R, Piccart-Gebhart M (2017). Adenoid cystic carcinoma of the breast – an aggressive presentation with pulmonary, kidney, and brain metastases: a case report. J Med Case Rep.

[52] Chen QX, Li JJ, Wang XX (2016). Similar outcomes between adenoid cystic carcinoma of the breast and invasive ductal carcinoma : a population-based study from the SEER 18 database. Oncotarget.

[53] Kulkarni N, Pezzi CM, Greif JM, Suzanne Klimberg V, Bailey L, Korourian S (2013). Rare breast cancer: 933 adenoid cystic carcinomas from the national cancer data base. Ann Surg Oncol.

[54] Martelotto LG, De Filippo MR, Ng CK, Natrajan R, Fuhrmann L, Cyrta J (2015). Genomic landscape of adenoid cystic carcinoma of the breast. J Pathol.

