# Supplementary material for: Histologic subtyping affecting outcome of triple negative breast cancer: a large Sardinian population-based analysis
Source: BMC Cancer. 2020 Jun 2;20:491. doi: 10.1186/s12885-020-06998-9 (PMC7268380; doi:10.1186/s12885-020-06998-9)

**Additional file 1**

**Supplementary Figure 1** Violin plot of tumor size distribution according to “Triple Negative” breast cancer histologic types.

IQR: Interquartile range.

**Supplementary Table 1** Correlation between tumor size and number of metastatic lymph nodes in different histologic types of “Triple Negative” breast cancer. Sardinia, Italy 1994-2015.

| Histologic type | # of patients | Spearman correlation coefficient | p value* |
| --- | --- | --- | --- |
| IBC-NST | 744 | 0.100 | 0.058 |
| Lobular | 62 | 0.005 | 0.974 |
| Adenoid cystic | 6 | - | - |
| Apocrine | 43 | 0.507 | **0.031** |
| Medullary | 39 | -0.052 | 0.809 |
| Metaplastic | 46 | 0.384 | **0.039** |
| Other | 27 | 0.458 | 0.133 |
| Total | 967 | 0.113 | **0.013** |

*The p-value are bold where they are less than or equal to the significance level of 0.05

**Supplementary Figure 2** Correlation between tumor size and number of positive lymph nodes in “Triple Negative” breast cancer histologic types. A linear regression model constructs the regression lines. The slope of the regression line symbolizes the average increase in the number of metastatic lymph nodes for each millimeter increase in tumor size for each histologic TNBC types, indicative of the influence of tumor size on number of metastatic lymph nodes.


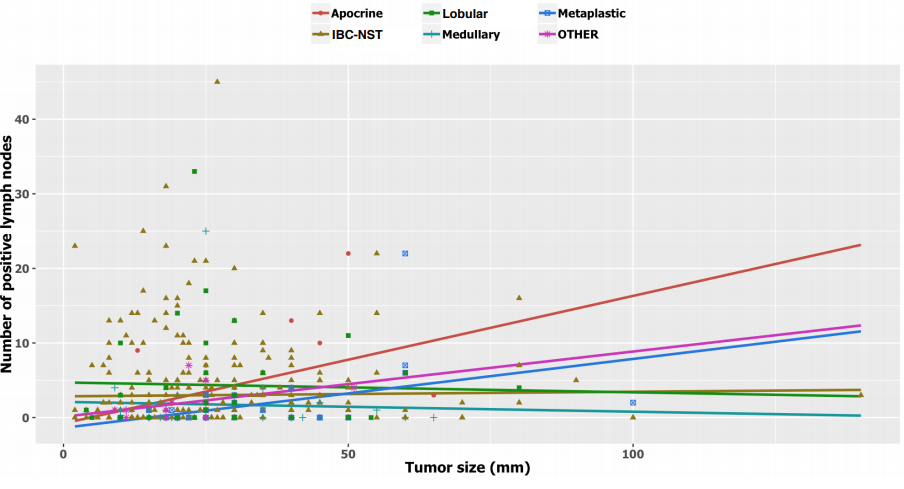

Supplement: Supplementary file 1 — Additional file 1: Figure S1. Violin plot of tumor size distribution according to “Triple Negative” breast cancer histologic types. Table S1. Correlation between tumor size and number of metastatic lymph nodes in different histologic types of “Triple Negative” breast cancer. Sardinia, Italy 1994–2015. Figure S2. Correlation between tumor size and number of positive lymph nodes in “Triple Negative” breast cancer histologic types. A linear regression model constructs the regression lines. The slope of the regression line symbolizes the average increase in the number of metastatic lymph nodes for each millimeter increase in tumor size for each histologic TNBC types, indicative of the influence of tumor size on number of metastatic lymph nodes. [file 12885_2020_6998_MOESM1_ESM.docx]
